# Critical assessment of refugees’ needs in post-emergency context: the case of Malian war refugees settled in Northern Burkina Faso

**DOI:** 10.1186/s12914-018-0176-0

**Published:** 2018-09-21

**Authors:** Idrissa Beogo, Amadou Darboe, A. Oluwafunmilade Adesanya, Bomar Mendez Rojas

**Affiliations:** 1 Centre de recherche en gestion des services de santé, FSA/UL-CHU de Québec; FSA/UL-IUCPQ; Faculté des sciences de l’administration, Université Laval, Pavillon Palasis-Prince, Université Laval, Québec (Qc), 2325 Rue de la Terrasse, Québec, G1V 0A6 Canada; 2 École Nationale de Santé Publique, Ouagadougou, 03 BP 7002 Burkina Faso; 3 University of Melbourne, Parkville, VIC Australia; 4 International College of Medicine, Institute of Public Health, International Health Program, National Yang Ming University, Taipei, Taiwan, Republic of China; 5 International College of Medicine, Institute of Public Health, International Health Program, National Yang Ming University, Taipei, Taiwan, Republic of China

**Keywords:** Critical assessment, Mali, War, Refugee, Humanitarian organizations, Burkina Faso

## Abstract

**Background:**

Empirically assessing the needs of refugees in camps is critical to the improvement of existing policies and programs that aim at enhancing their well-being. By neglecting the needs of refugees, interventions may fail to capture the complex patterns of refugees’ daily lives within camps. This paper provides a comprehensive assessment of the needs of encamped Malian refugees in Northern Burkina Faso following the 2012-armed conflict. In addition to assessing the needs of Malian refugees, the study aimed to critically assess from an upstream perspective the degree of their involvement in policies and practices that are targeted towards improving their livelihood.

**Methods:**

We took an “upstream” view on the lives of Malian refugees to identify their unmet needs. A purposive sampling strategy was employed to collect data from various media sources, including data aggregated from the website of the United Nations High Commissioner for Refugees (UNHCR). The most populous refugee camp (Mentao) was visited in September 2012 and in-depth group discussion and interviews were conducted with key informants, including nine camp representatives and four officials from the central and decentralized administrations.

**Results:**

Media canvass combined with the UNHCR level 2 census revealed a flawed headcount of refugees, which was 205.4% higher than the real number in Burkina Faso. Although refugees live harmoniously with the natives and their security has been assured, they strongly complained about the number of unused food items distributed. Camps were distributed among humanitarian organizations leading to differential advantage and resources from one camp to another. Additionally, idleness, lack of classrooms facilities for pre-school children and lack of continuous healthcare services were major concerns raised. Further, refugees expressed limited involvement in the planning and implementation of programs that are related to their welfare.

**Conclusion:**

This study revealed that refugees’ voices were not taken into consideration in making tailor-made programs. This calls for more comprehensive surge capacity to deal with refugees’ basic needs. Further, a strong leadership from hoststate should be encouraged to offer equal opportunities to refugees regardless of their camps. Finally, an innovative strategy is needed to build a reliable database that could enhance the design, implementation, monitoring and evaluation of policies and programs.

## Background

At the end of 2015, the United Nations High Commissioner for Refugees (UNHCR) reported that 65.3 million people worldwide were forcibly displaced and 21.3 million (33%) of them were refugees fleeing from conflicts and persecution [1]. The number of refugees is on the rise as countries continue experiencing violence and political instability resulting in (or as a result of) intra-state clashes (civil wars, armed conflicts) [2], such as the one experienced by the Republic of Mali in 2012. Indeed, the human mobility has been omnipresent [3], including international migration [4] (voluntary or economic [5] or forced [4]). But the refugee status is well defined and protected under the 1951 United Nations (UN) convention and its 1967 protocol [6]. Low-income-countries (LICs) remain the top providers of refugees but at the same time, 90% settle in these countries [7]. Contrary to the pervasive opinion about refugee settlements, close to 30% of the global refugee population was hosted in Africa and about half of the global refugee population are under the age of 18 [1].

The consequences of inter or intra-state political unrests and armed conflicts are enormous for all those involved [2]; especially for women, children, elderly and frail persons. Host countries (or regions) bear the burden of having to respond to the unpredicted basic needs of newcomers. These include but are not limited to the need for healthcare, food, shelter, employment, and security. In the context of LICs responding to the healthcare needs of refugees puts the already fragile health systems under immense pressure [8]. Furthermore, a protracted stay of refugees in host countries, especially low-income ones, is likely to have some long-term socio-economic and environmental consequences [9, 10].

Refuge-seeking is fraught with precarity [11] which includes cohabitation challenges with host neighbors and welfare constraints [12–14]. Running away from war and persecution can cause people to lose connection with loved ones, weaken social capital, loss of homes, properties, and employment, which results in constant anxiety, anger and depression [15–17]. Life-threatening experiences can be very traumatizing with a propensity to negatively impact the mental, physical and social well-being of refugees [15, 18]. Aside from psychological impairments, refugees are confronted with some socio-economic challenges, which exacerbate stressful experiences during settlement [19]. It is becoming increasingly recognized that policy and psychosocial interventions in host countries play a critical role in the adjustment and integration process of refugees [18, 20, 21]. A systematic review has contended the effectiveness of school-based interventions in reducing psychological disorders in refugee and asylum-seeking children [20].

Being aware of challenges encountered by refugees in host countries, the World Health Organization and the International Organization for Migration jointly encourage governments to take stock of strategies and resources at their disposal to ensure better health and well-being for migrants [3]. Such a commitment is in line with the UN Declaration in 1986 on the Right to Development [22]; thus, affirming the right of every individual ─and refugees being no exception─ to participate, contribute, and enjoy economic, social, cultural, and political development. Since participation is the sine qua non of achieving program goals, thus refugee participation is highly critical for the successful design, implementation, evaluation, and sustainability of targeted interventions [22]. Rempel [23] demonstrated how the United Nations Relief and Works Agency for Palestine Refugees in the Near East succeeded in participatory projects in which protracted Palestinian refugees are involved from the planning to implementation and evaluation.

Unfortunately, as some authors [24, 25] put it, refugees appear to be entirely subjected to unilateral aid regimes and the protection provided by international organizations, with very little choice of changing their fate. Campbell [26] challenged the official position and the common perception that non-encamped refugees are an economic burden. Using the case of the refugees in Nairobi, he contended that local integration is a possible solution to protracted exile although it raises important issues for their protection. In a similar vein, Crea et al. [27] compared urban and encamped refugees and asserted that the urban refugees reported significantly higher satisfaction with overall physical health and social well-being than their encamped counterparts. For humanitarian actors and above all host governments, encampment favors centralizing of distribution outlets, which remains the most effective way to allot resources, and is deemed appropriate to rule out security threats [28]. For the latter, recent facts surprisingly indicated that refugee camps are used as Trojan horse for terrorism ―e.g., terrorists who killed the 67 persons in West Gate Mall of Nairobi (Sept. 21, 2013) have reportedly stayed in Dadaab camp, likewise the perpetrators of the killing of the 148 students in the Garisha University College, Kenya (Apr. 02, 2015).

Although refugee issue is ubiquitous, studies examining their livelihood and needs from an ‘upstream perspective’ are rare. The upstream perspective considers critical questions about the projects developed for the refugees such as: For what? What refugees want? What is the ultimate goal of the donors and the host State? How is the success measured? The upstream perspective also considers both (i) beneficiaries involvement in projects thought for them and (ii) distal factors —beyond individual level— such as socio-political factors (e.g. government policies and programs) that affect the lives of refugees in host countries. Hence, using an upstream approach, this paper takes a closer look at the situation of Malian war refugees who fled to Burkina Faso as a result of the 2012-armed conflict. The civil war was a collateral effect of the *Arab Spring*. The latent Azawad independence claim surfaced with the reinforcement of the Touareg separatist group by the foreign legion (formed by Touaregs) in the Libyan army dismantled by the international coalition. They were backed by Islamist groups ―linked to AlQaeda or its mainstream― and have sparked Malian chronic political conundrums that have resulted in a coup d’État perpetrated by an ephemeral Junta. The suddenness of its outbreak in January 2012 and the rapid progression of insurgent groups sent thousands of inhabitants into exile. Some were internally displaced while others crossed the border to become refugees in neighboring countries. People fleeing to Burkina Faso were considered refugees based on prima facie as described in the literature [29, 30].

To our knowledge, few studies have examined the daily lives of refugees, their needs, and prospect in non-emergency context, as well as their level of participation in the development of humanitarian policies and programs. A number of studies have provided information on social protest between refugees and camp administrators and/or local authorities [31, 32]. However, from the upstream standpoint, little is known in regard to refugee participation in policies and programs that matter to their welfare. Such participation —if it exists— is fundamental for more sustainable and responsive projects [23, 33]. Those who have looked inside refugee camps have traditionally focused on protest for self-governing [32, 34, 35]. Hence, researchers have overlooked the powerful benefits of refugees’ voice in policy and program development processes.

Faced with this literature gap, the present study attempts to evaluate the needs of Malian war refugees’ settled in Burkina Faso. The novelty of the paper is that it in addition to assessing refugees’ needs, it also investigated the extent of their participation in the planning and implementation of policies and interventions targeting their welfare.

The varied group of actors referred to in this article as ‘humanitarian organizations (HOs)’ consist of the supranational organizations (e.g. UNHCR, United Nations Children’s), varied non-governmental organizations (NGOs), that principally responds to emergencies in the field.

## Methods

### Setting

We narrowed the study focus to Mentao camp, the most populous camp in the country (6000 persons officially registered at the time of the study), located at 10 km from Djibo (province of Soum), on the highway No. 23, between Ouahigouya and Djibo. The refugees settled in Burkina Faso belong to various ethnic groups: Touaregs (76%), Arabs (10%); the rest include Fulani and Songhai, to cite few [36]. They hail from both towns and villages of the Republic of Mali and have diverse socioeconomic backgrounds (e.g., teachers, pastoralists, farmers, and traders).

#### Participants and data collection

In September 2012, we conducted on-site discussions with the Mentao refugee camp representatives committee. Participants were interviewed on tangible and intangible dimensions of their daily lives (Table 1) adopting the approach used by Ito et al. [37].

**Table 1:** Themes approached with Mentao camp refugee representatives

Intangibles themes	Question items	Tangibles themes	Question items
Occupation	- Daily business of residents - Residents’ former occupation - Job opportunities	Camp infrastructures (tents, space, common areas…)	- Residents’ opinion on: • Shelter versus family size, intimacy • Life setting • Security
Healthcare	- Specific program benefitted (eg.: vaccination) - Benefits of a routine vaccination program - Other programs: • Curative • Emergency • Reproductive • Specific groups (women, children, elderly) - Physical access (distance, time) and financing mechanism	Water and sanitation	- Physical accessibility - Perceive quality and quantity supplied
Participation in projects developed for the camp	- Role of the committee in food items distribution - Formal existence and regularity of meeting between humanitarian organizations (HOs) & camp representatives committee	Food items and cooking	- Supply interval of foods items - Itemized list of foods items distributed - Quantity & quality of foods items distributed - Other services provided
Relationship with neighborhood	- Within camp neighbor - Within camp culture and people - Camp site neighbor - Existence of conflict resolution mechanism	Education & Entertainment	- Children education program - Entertainment program
Official (politic, aid agencies) visits	-Refugee status proceedings with the United Nations High Commissioner for Refugees - Domestic, National & international authorities field visit - HOs field visit	–	–

Because the study intended to capture feedback on experiences of refugees, including sensitive issues that participants may not feel comfortable sharing in a certain context, we combined purposefully three techniques to gather the data. First, we screened media released information starting from the conflict outbreak in the Republic of Mali. We particularly emphasized on information regarding refugee population movement and distribution in Western Africa; where they settled, their headcount, living conditions, and actors intervening on the field, including the host governments’ contribution. Media information included aggregated data from UNHCR’s website and officially released newspapers. Second, health-related data were gathered from the department of health and from UNHCR released. This was intended to figure out their health status. Finally, Mantao camp was selected for an on-site visit, followed by an in-depth group discussion with the camp representatives.

### Interview session

The PI worked very closely with the local welfare department whose staff was well acquainted with the camp and its representatives. For trust consideration, they served as a liaison between the refugees and the PI to organize the meeting. This was necessary, as, at the time, there was diminishing trust between the camp residents and HOs, especially the UNHCR.

The interviewer, (IB) was lucky enough to meet the representatives who were reluctant but at the same time interested in the tangible outcome of our meeting. They argue that they (representatives) are on daily basis questioned but their (refugees) fate has not changed. The interviewer befriended them and declare his position as follows: “*I am [Name], a public health doctoral student at National Yang Ming University, Taiwan, and would like to conduct a critical need assessment and sketch up your living conditions, for the purpose of sharing findings with scholars and policymakers.”*

To maximize the opportunity of having refugees voice out their experiences, including sensitive ones, we purposefully combined three techniques. First, we concomitantly conducted a group discussion with naturalistic observation [38], which lasted for 2h45mn. But instead of two discussion sessions as planned (with males and females), we conducted only one as we were unable to obtain the consent of husbands to meet the females. Nevertheless, the entire committee composed of nine persons ―all males― participated.

Apart from socio-demographic characteristics, the group interview emphasized on the discovery of participants’ views, voices, feelings, and experiences. Consequently, it enabled the participants rather than the researcher to lead and shape the course of the discussion. The naturalistic observation allows first, the observation of behaviour in the real world that ensures the ecological validity. Because other refugees were not aware of our presence, we hypothesized this observation is free of a behavioural bias. Second, it helps establish the external validity, corroborating information stemmed from the group interview. Lastly, ethically, naturalistic observation prevents manipulation of certain variables or facts that may naturally occur.

During the process, we took the opportunity to observe existing infrastructures, discussed how they settled in the camp, gathered information about their living conditions in terms of their; physical security, access to health care, and involvement in administrative decisions affecting their daily lives. A semi-structured questionnaire was developed purposefully, addressing all the themes listed in Table 1.

Finally, formal discussions were carried out with other key informants, that included; Djibo District Health Director (35 min) and welfare department staff (45 min), Department of Health representative (20 min), and one NGO representative (15 min). The raw data and the qualitative comments were collated. Qualitative comments were thematically analyzed. Inclusively, administrative preparation and data collection took the entire month of September 2012.

## Results

We examined web reports provided by the UNHCR and NGOs, gathering information about camp characteristics (location, population, and ethnic composition). Data collection on healthcare were particularly challenging because all the three NGOs, Doctors of the World (DoW), Doctors without Border (DwB), Terres des Hommes (TdH) refused to provide their data, regardless of the official request we presented. The first argued that the request should be addressed to their main headquarters ―at Geneva―, while DwB directed us to the DoH, stating all data are sent there, and TdH did not get back despite numerous recall emails.

### Settling proceedings and the flaws in refugee headcount

**Fig. 1:**
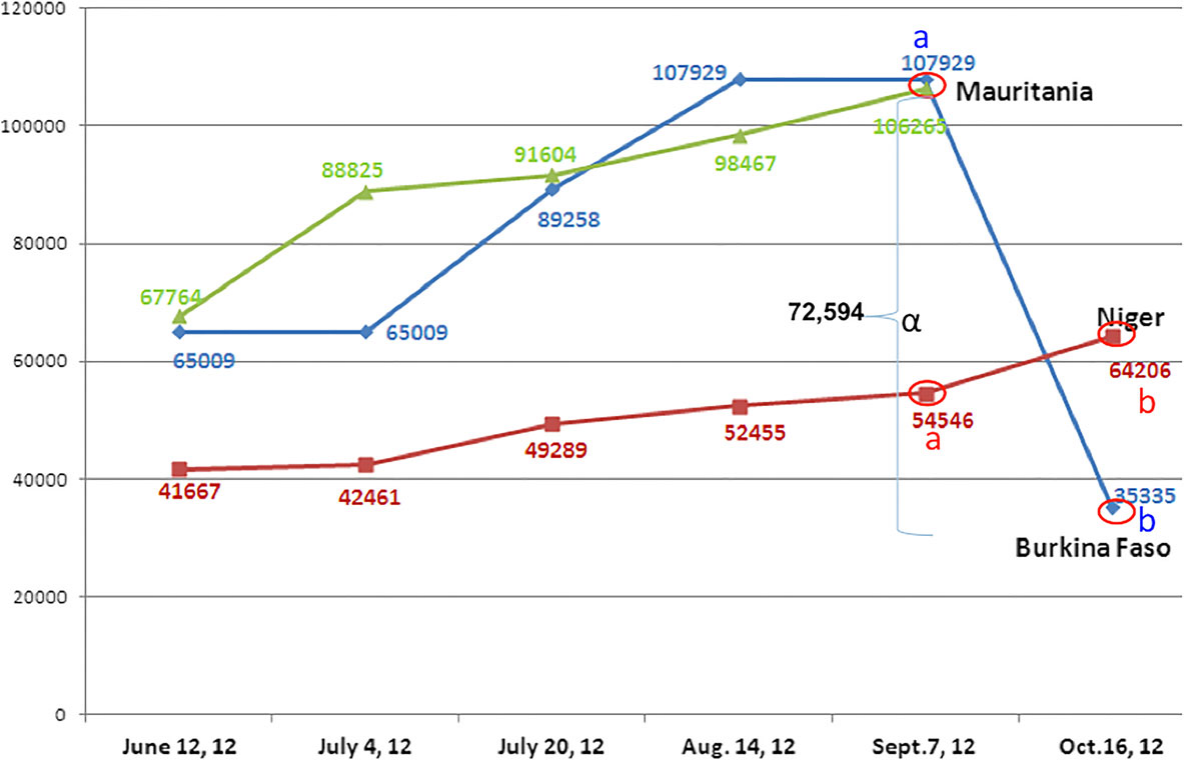
Evolution of Malian refugees count in the major host countries. a Official figure before level 2 census release. b Refugees population from Level 2 census. α: Difference between the refugees official count and the real population (level 2 census). Source: Compile from UNHCR (http://www.unhcr.org/cgi-bin/texis/vtx/search?page=search&query=malians+refugies+in+Niger&x=0&y=0)

From media released, interviews of UNHCR domestic representative and key informants, the refugees count trial was done in two rounds. Level 1; census conducted at the first point of contact with the host country, consisted taking the identity of the household’s head and the household size. Then, a UNHCR card is given weeks later. The Level 2 census is relatively more exhaustive. All the refugees from all individual households were registered and information on socio-demographic characteristics: household size, gender, age, marital status, etc. were recorded. By September 2012, the present study synthesized data obtained from level 2 census. It revealed that the actual number of Malian refugees hosted by Burkina Faso is three times (35,335 persons) less than the official figure (107,929 persons) (Fig. 1). Our canvass of newspaper to trace the refugee headcount indicated that the HOs have for long dealt with these flaws in headcount. For instance, in an interview given to a local newspaper, “Le Pays” on July 12, 2012, the UNHCR country representative had said: *“The level 1 census that is done, ended up with 65,000 refugees. We are preparing for the level 2…that operation will give us valid figures and we could give them food ration cards. Later on, the government would give them refugee cards*….”[translated by the PI]. At the same time, UNHCR website reported on July 12, 2012, that 107,929 refugees were hosted by Burkina Faso. In another local newspaper, “Sidwaya” of September 3, 2012, the same Burkina Faso UNHCR representative mentioned as we quote: “*Today, we are at 90,000 persons…I would say that, theoretically, the figure of 100,000 is reached….*” [translated by the PI]. These figures are 205.4% higher than the real population in the camps (see Fig. 1). This fact contrasts with the insufficiency and the irregularity of food items ration, as reported by refugees. Second, the upward miscount of the refugee population hinders the HOs’ performance as for the reliability of their planning, forecasting, and assessment of food and non-food items, staff recruitment, and infrastructure implementation, to cite a few. Finally, a biased denominator may refrain donors from financing efforts.

Informally, we were advice by a high ranked staff from one of the very active partner HOs that UNHCR was using erroneous headcount for its operations. Based on their own field census ―in the camp they are in charge―, officially reported data were far higher.

### HOs organization in the field

It appears that in every camp, health programs (and education to a certain extent) were fully managed by a specific HO. At the time of the study some camps had a healthcare service, but Mentao, on the other hand, was relying on Djibo health services (situated ten kilometers away). HOs offering health services were mainly working alone per camp (e.g., DWB alone in Gandafaou and Ferrerio [39, 40]. In contrast, those working in other sectors (e.g., water, sanitation, education …) were many per camp (Table 2). By September 2012, some NGOs were offering 24-h in-camp health services while others were providing outreach services. Services given differ from one NGO to another in the same sector. In addition to HOs, the government has organized a special vaccination campaign against some epidemic-prone infectious diseases namely; meningitis, measles, and poliomyelitis.

**Table 2:** Example of the numerosity of actors with the same expertise per camp

Province (Camp site)	Partner
Non-food items	
Kadiogo (Somgandé)	Terre des Hommes, Red Cross
Houet	Red Cross
Sourou (Tougan)	Terre des Hommes
Soum (Djibo, Mentao, Damba)	Plan Burkina, Red Cross, UNESCO, Oxfam, Hope 87
Oudalan (Fererio, Déou, Bibissi, Gandafabou, Gountour Gne Gne)	CICR, CRS, Red Cross, OCADES, Caritas, Plan Burkina, Oxfam, Help
Education	
Fererio	UNICEF, Terre des Hommes, FDC/A2N, NRC, Croix Rouge
Gandafabou	UNICEF, FDC/A2N, Save The Children (Japan & Canada), NRC, Plan Burkina, Red Cross
Mentao	Plan Burkina, UNICEF
Damba	Plan Burkina, UNICEF
Ouagadougou	UNICEF, TDH, CREDO
Bobo Dioulasso	UNICEF, TDH, CREDO

### Challenges faced by refugees in their daily life in the camp

Refugees representatives were very grateful to Burkina Faso for its welcoming and hosting without any segregation. They stated that: “*Newcomers are still reported in this camp…. It may be an indication that our refuge site is quite safe and perhaps offers livable commodities.*” [translated by the PI]. However, they reported that their daily life is fraught with basic challenges. The monthly food ration includes 12 Kilograms of rice, 900 g of bean, and 0.15 l of cooking oil per individual, but the service does not include condiments (vegetable). Finally, refugees have experienced delays of 14 to 45 days in the food supply and were served with food items that do not fit with their dietary habit. One said: “*We are not used to must stuffs served…bean is not part of our dietary culture….*”[translated by the PI], and we have vainly proposed alternative solutions: 1) adding meat, milk, and condiments; or 2) replacing beans with some cash for vegetable, milk, and meat; or 3) full cash payments. As a result, most of the refugees solicit financial help from relatives in the Diaspora arguing that the given food items expose infants to malnutrition.

Another issue raised by key informants was related to the design of camps. Tents are aligned in dense rows and columns, which in their opinion do not correspond to the Touareg people’s habitat. It is felt to be very overcrowded, with a common block of toilet/latrines and was considered to render people vulnerable, particularly women. The toilets in refugee camps are not separated by gender, have no lighting and no locking mechanism. Other issues were related to drinking water. During our data collection tours, we observed that the existing water tower —at the time of the survey— was functioning for only two weeks. Although critical to thousands of refugees, it took eight months for residents to enjoy the potable water. Finally, the participants highlighted the hardship in getting health care, including the distance to Djibo, the difference in the system, or the cultural issues in their interaction with health personnel.

The results of the present study note that idleness of adult refugees remains a noticeable issue. For the representatives of Mantao camp refugees, many of them were civil servants or private entrepreneurs in their home country and do have the expertise to help in certain aspects of humanitarian projects. Several propositions in that sense have been made, including resuming study classes with Malian education program for children, but they received a formal disagreement. *“Some of us are teachers and could help go on teaching Malian education program to our kids....”* said one; another one added: “*We were warned that the project of recruiting local teachers has failed [….] we were asked to send our kids to local schools ―Burkina Faso education program. It is a recommencement….”* [translated by the PI]. Their relative emancipation in electing a representative, as reported elsewhere [31], was not sufficient to obtain a positive response to their suggestion for food ration or camp organization. Apart from their professional background, one participant said: “*our committee asked them* [HOs, UNHCR included] *to be involved in in-camp services such as the monthly food distribution, in order to get a small pay. That resolves our unemployment status and adds to the quality of our life.” *[translated by the PI].

For local administration, refugees have the benefit of health insurance package and feel that they are hard to please, always complaining. However, Djibo health officials have observed that refugees once ailed, behave disrespectfully, in always wanting to be seen by the health personnel (Nurses and doctors) in urgency, shunting the waiting line. As for the local welfare officers, in addition to not being allocated a budget, they complained to be busy with repetitive meetings with no real added value in the outcome for the refugees. Furthermore, they have noticed a black market. The days following food items distribution, refugees barter or market foods items distributed, but it has not been confirmed by the key informants.

## Discussion

In this study, we assessed the needs of Malian war refugees living in refugee camps in Mentao, a community situated in the northern part of Burkina Faso. As a needs assessment study, we paid an utmost attention to the concerns of refugees, the type of activities in place to facilitate their settlement, and the extent of their involvement in those activities. We further teased out the varied group of actors (HOs) working with the refugees. Collectively, the study laid out three crucial findings.

First, despite the media attraction, the daily life of refugees is fraught with unmet basic needs. Malian refugees settled in Northern Burkina Faso attracted great attention from the media, politicians of both sides (Burkina Faso and Mali), even from UNHCR white collars, researchers as well as NGOs that continue to compete for space to be visible in action. Although the camp representatives are overwhelmingly solicited and consulted by the UNHCR and other HOs, little room has been given to their voice in programs development, letting them rely entirely on aid and HOs. For instance, continuing serving them with food items that do not meet their cultural dietary habit for almost a year after their settlement. Consequently, residents are compelled to unfavourably swap in black market the food items served in exchange for milk or meat that fits their tradition. Hence, this deepens their vulnerability. Potable water, as well as healthcare services, have been among the basic needs for which, much time has elapsed before Mentao refugees got acceptable programmes. These are some of the salient and erratic facts that question the host State government and particularly the UNHCR’s expertise on their preparedness and the quality of their responses to refugees needs over the emergency stage. This is especially true when the programs are run with inflated refugee headcount. Heavy bureaucracy, lack of proactiveness or strategic choice of HOs, or ignoring domestic expertise for a synergy between emic and etic views [41], are to our belief associated to failure to prevent the complex social makeup to rule out some groups marginalization, as well as ensure their participation in decision-making processes. Thus, the deficit in comprehending the within and between ethnic group hierarchal social relations have ended up in a clash between ethnic groups, dividing the camp into two (Mentao North and South). As one member of the groups said, “*Even in heaven, some people could not mix”* meaning some groups must not share the same living space and facilities with others. This must be known by HO’s experts. We argued that, because refugees already got a host country, their issue was relinquished to the second plan in order to focus on the peace-building path in their home country. But Mentao refugees worried more about their immediate vital needs to survive. Returning remains an aim but because of the painful memories and the lingering fear of persecution and insecurity, waiting for a total calm back home is a necessity. Nevertheless, of the available solutions identified in refugee literature [35], the present study participants did not mention the resettlement in other countries as a possible pathway.

Second, from an upstream view, apart from the issue of excluding refugees in project development and implementation, we found dissimilarities in projects run between camps. For instance, while by August 31, 2012, education remedial is ongoing for students, in Fererio (by TdH), Gandafabou (Save the Children) and Damba (PLAN Burkina), things were on standby in the most populous camp of Mentao [42], keeping them in a limbo. By September 2012, DwB was running in-camp health clinics in Gandafabou and Férrerio and weekly mobile clinics in Dibissi, Ngatoutou-Niénié, Déou for informal settlements [39]. However, there were no continuous healthcare delivery services in Mentao camp (the most populous). Overall, the fate of refugees differs from one camp to another, depending on which HO intervenes, services given may exist or not, or even delivered differently. We argue that these differences in running projects may potentially be due to the financial wealth of HOs. Those with more financial capabilities may tend to work in solo and be alone accountable for the project achievement. This eventually deprives refugees the opportunity to benefit from other valuable expertise. On the other hand, the multiplication of HOs in a single camp may be due to their lesser financial weight yet, looking for visibility in the arena. Nevertheless, concerning the high concentration of HOs in a single camp, Riddell [43] found a diminishing marginal return while the number of NGOs increases. This difference in care, and services offered, or in running projects unveiled by our study suggests a lack of a national framework. Our study suggests that the UNHCR has exhibited a weak capacity to effectively assume its leadership and coordination role to respond at the emergency phase. This has led to inefficient cooperation, which may further give room to a lack of accountability from NGOs. Duplication was observed in data collection. For instance, while HOs claim sharing data with Burkina Faso DoH (not confirmed), this latter in parallel has carried out surveys in March and July 2012 in refugee camps. This absence of ‘talking to each other’, raises concerns as for whether a National Commission for Refugees does exist, and the HOs are a member of its permanent secretary. A salient indication of aforementioned unpreparedness is pictured by the prevalence of malaria morbidity of 50 to 60% at week 34, in the main camps (Fererio, Gandafabou, Damba, and Mentao) [42]. The flaws in management ending up with the wrong count of refugees, misleading the public opinion, as exactness of the population is the backbone of all intervention planning, management and advocacy. Our view is concordant with Oxfam comments concluding that HOs staff had limited experience in refugee emergencies and were confused about UNHCR’s role [44]. Previous writers in the NGO literature, such as David Korten [Quoted by 42: 419] observed that NGOs often had difficulties working together because of jealousy that paralyzes the achievement of shared purposes. Many of them strive to preserve their freedom in choosing where and with who to work with. This attitude poses critical concerns in terms of social justice, for instance, equity in access to services among refugees, as some groups were more fortunate than others.

Finally, we could avow a hidden battle among HO for more visibility in the field, hindering the achievement of the core aims of refugee well-being. Hence, despite multiple structured meetings from top to local level, the response to refugees’ needs was far from appropriate. Information we had with various key informants, yielded a contrasting agreement between the official statement and field intervention versus the realities of life in camps (e.g., refugee number and food items). Every HO seems to act on their own volition as it suits their personal agenda. For instance, altogether, none of the HOs approached had accepted an interview or agreed to provide data. Overall, it appears that different camps were “shared” between HOs, what highlights an absence of a commonly built national framework to abide with. ‘How could it be?’ As Turner put it, refugees are asked to have a lower profile, to be human: with no political voice [45]. Besides, as Mentao camp representatives stated, they were overwhelmingly solicited by HOs to whom they repeatedly voice their hardship. But, who should take a step in the resolution of their situation when there are multiple actors, lack of clear leadership? Undoubtedly NGOs’ survival is significantly associated with the capacity to successfully campaign to raise adequate funds, therefore, only outcomes reflecting their intervention matter to them. UN agencies have since the 1940s ―following World War II― involved NGOs in policymaking [46]. Nevertheless, Riddell’s landmark book illustrates the phobia of most NGOs to put forward to the public information on their project, and when it is done, “it is heavily biased toward showing success…” [43].

## Conclusion

This study is one of the first to address issues surrounding Malian war refugees. Hence, we took an upstream view of their needs assessment and the HOs’ preparedness and organization for field response. Our study underscores how the first year of a possible protracted refugees settlement appears very to be challenging and showed their resilience to survive at the time of international mobilization of resources. The study revealed that contrary to official position and the popular opinion, Malian refugees are not without basic needs. Apart from the visible lack of preparedness for emergency response, post emergency response scheme has not provided any means of involvement for refugees in programs development and /or implementation. They are still expected to eke out their existence by themselves and fall into a state of dependency on humanitarian and other external aid, with the risk for some being recruited by armed groups ―that the sub-region is rife of―, for financial gain. Finally, since Northern Mali’s sociopolitical concern has been recurrent since the 1960s ―1960, 1963, 1990, 2006, and 2012―, structural reform policies are needed to address subsequent consequences, including refugee issues.

## Abbreviations

DoH: Department of health
DwB: Doctor without border
HO: Humanitarian organization
LMIC: middle income countries
NGO: Non-governmental organization
PI: Principal investigator
TdH: Terres des Hommes
UN: United Nations
